# Anti-Inflammatory and Anti-Fibrotic Effects of a Mixture of Polyphenols Extracted from “Navelina” Orange in Human Hepa-RG and LX-2 Cells Mediated by Cannabinoid Receptor 2

**DOI:** 10.3390/ijms26020512

**Published:** 2025-01-09

**Authors:** Ilenia Saponara, Emanuela Aloisio Caruso, Miriam Cofano, Valentina De Nunzio, Giuliano Pinto, Matteo Centonze, Maria Notarnicola

**Affiliations:** Laboratory of Nutritional Biochemistry, National Institute of Gastroenterology IRCCS “Saverio de Bellis”, 70013 Castellana Grotte, Italy; ilenia.saponara@irccsdebellis.it (I.S.); emanuela.caruso@irccsdebellis.it (E.A.C.); miriam.cofano@irccsdebellis.it (M.C.); valentina.denunzio@irccsdebellis.it (V.D.N.); giuliano.pinto@irccsdebellis.it (G.P.); matteo.centonze@irccsdebellis.it (M.C.)

**Keywords:** hesperidin, Navelina orange extract, liver inflammation, fibrosis, CB2R, p38 MAPK, COX-2, TGF-β

## Abstract

Navelina oranges (*Citrus sinensis*) are rich in phytonutrients and bioactive compounds, especially flavonoids like hesperidin. This study investigates the anti-inflammatory and anti-fibrotic properties of hesperidin (HE) and a polyphenol mixture from Navelina oranges (OE) in human hepatocytes (Hepa-RG) and hepatic stellate cells (LX-2), in order to elucidate the underlying molecular mechanisms. In Hepa-RG cells, HE treatment increased expression of cannabinoid receptor 2 (CB2R), which was associated with down-regulation of p38 mitogen-activated protein kinases (p38 MAPK) but had minimal impact on cyclooxygenase-2 (COX-2) and transforming growth factor-β (TGF-β) levels. Conversely, OE treatment not only enhanced CB2R levels and reduced p38 MAPK, but also promoted a significant reduction in both COX-2 and TGF-β levels, suggesting that OE might be more effective in mitigating inflammatory and fibrotic processes than HE. In LX-2 cells, HE treatment caused a notable decrease in both COX-2 and TGF-β levels, reflecting its efficacy in targeting fibrosis-associated inflammation. OE treatment, on the other hand, reduced Nuclear Factor-Kappa B p65 (NF-κB) expression, a critical transcription factor involved in inflammatory responses, though it did not significantly affect COX-2. LX-2 cells induced to fibrosis with TGF-β and treated with HE and OE showed a reduction in the expression levels of several fibrosis markers. In addition, HE and OE showed antioxidant effects by increasing protein levels of Cu, Zn superoxide dismutase (SOD1), Mn superoxide dismutase (SOD2) and catalase (CAT) and influencing the state of lipid peroxidation. Further research is needed to explore the effects of the treatments in activated hepatic stellate cells and in vivo liver disease models.

## 1. Introduction

Navelina oranges, botanically classified as *Citrus sinensis* species and belonging to the *Rutaceae* family, are native to Brazil and now widely cultivated throughout the Mediterranean region [1]. Orange fruit processing, especially in the production of juices and citrus-based beverages, generates a large amount of citrus by-products, such as peels and albedo (the white layer between the skin and the pulp of citrus fruits), which contain high concentrations of health-promoting phytonutrients and bioactive compounds including flavonoids, phenolic compounds, vitamins, dietary fibers, and essential oils [2,3,4,5]. Due to their biological activities, these citrus by-products can be employed as nutraceuticals and functional dietary supplements, with growing interest in the pharmaceutical field [6,7,8].

In Navelina oranges, the hesperidin is the primary representative among key flavonoids, accompanied by others such as naringin, naringenin, nobiletin, and tangeretin [9]. Overall, these flavonoids are well-known for their antioxidant properties [10], cardiovascular support [11], anti-inflammatory effects [12] and potential anticancer properties [13] making them promising nutraceutical agents for handling various pathological conditions.

Numerous in vitro and in vivo studies investigated the anti-inflammatory effects of HE and its metabolites, although the results are affected by the experimental model and chemical form of the HE [14,15,16,17]. HE, at the concentrations of 20 and 30 μM, has been demonstrated to reduce NO₂ levels and suppress iNOS expression in LPS-stimulated RAW264.7 macrophages [18]. HE also reduced PGE₂ production with no influence on COX-2 protein levels [18,19]. Interestingly, it turns out that, the combined action of a mixture of polyphenols (nobiletin, naringin, and HE) from *Korean Citrus* aurantium significantly reduced COX-2 expression at both mRNA and protein levels, as well as the levels of pro-inflammatory cytokines [20]. The protective effects of HE against LPS-induced toxicity have been demonstrated in an in vivo study. HE significantly reduced oxidative stress by decreasing lipid peroxidation and increasing antioxidant molecules, particularly glutathione (GSH) and CAT [21]. The anti-inflammatory effects of hesperidin are primarily due to its antioxidant and free radical scavenging effects, which neutralize reactive oxygen species (ROS) and reduce oxidative stress. HE decreases lipid peroxidation and enhances the activity of key endogenous antioxidant enzymes, such as SOD and CAT [22,23]. In particular, SOD1 constitutes about 90% of total SOD activity. In turn SOD2 is located in the mitochondrial matrix. Both SOD1 and SOD2 bind superoxide and convert it into hydrogen peroxide [24,25]. Following the action of SOD, CAT further rapidly transforms hydrogen peroxide into water and oxygen, preventing its accumulation and subsequent conversion into more reactive species, such as hydroxyl radicals, which can exacerbate oxidative stress and lead to inflammatory responses [26].

Recently, our clinical study indicated that consumption of whole oranges for four weeks significantly reduced the prevalence of hepatic steatosis in subjects with metabolic dysfunction associated with steatotic liver disease (MASLD) compared with the healthy control group [27]. In this context the emerging role of CB2R in liver disease is increasingly recognized due to its significant protective effects against liver injury [28]. Primarily expressed in peripheral tissues, CB2R exhibits anti-inflammatory and anti-fibrotic properties [29]. CB2R modulates key intracellular signaling pathways, including the inhibition of adenylyl cyclase and activation of MAP kinase and PI3-kinase, which are crucial for regulating cell survival, inflammation, and fibrosis [30]. In vivo studies demonstrate that CB2R knock-out mice are more susceptible to hepatic damage, displaying increased liver inflammation and steatosis compared to wild- mice [31]. These findings highlight CB2R as a promising target for therapeutic interventions in liver diseases.

Considering the beneficial effects that flavonoids, a subclass of the polyphenol family, have on the liver, our study investigated the effects of HE (the main polyphenol detected in the orange fruit) and a mixture extracted from OE, on cultures of hepatocytes (Hepa-RG) and hepatic stellate cells (LX-2), involved in the fibrotic process of liver tissue.

The aim of this study is to determine whether HE and OE could mitigate liver injury through their anti-inflammatory, anti-fibrotic, and antioxidant properties, as well as to explore the molecular mechanisms underlying these effects. In particular, we will focus on the role of some key players in inflammation and fibrosis, such as p38 MAPK, NF-κB p65, COX-2 and TGF-β, and biomarkers involved in antioxidant effects, such as SOD1, SOD2 and CAT. We will also investigate the ability of these treatments to influence p38 MAPK activation, which induces the expression of factors involved in inflammation [32]. We will then assess whether HE and OE can modulate the expression of CB2R, a receptor with established protective functions in the liver.

## 2. Results

### 2.1. Analysis of OE Characteristics

**Figure 1:**
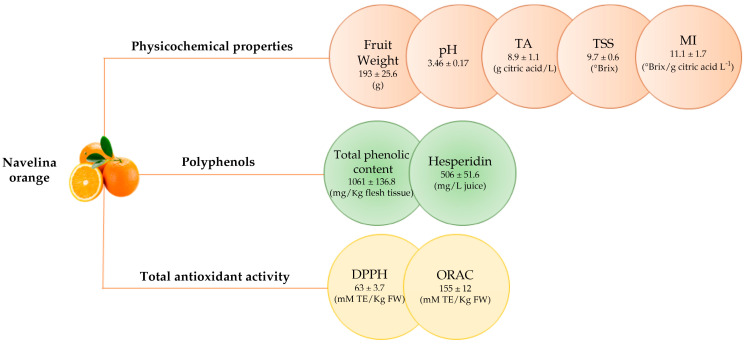
Physicochemical properties, polyphenols content and total antioxidant activity of Navelina orange. All data are reported as mean value ± standard deviation of six samples. TA: Titratable Acidity; TSS: total soluble solids; MI: maturity index; DPPH: 2,2-diphenyl-1-picrylhydrazyl test; ORAC: oxygen radical absorption capacity test.

The OE was analyzed to determine its physico-chemical characteristics, polyphenol content, and antioxidant activity. Figure 1 shows the mean values of the total phenolic content and hesperidin content of the six Navelina orange samples analyzed. The values are expressed as mg/kg flesh tissue and mg/L juice, respectively. Hence, the extract is rich in polyphenols, in particular hesperidin, its main component. The mean of total antioxidant activity measured by DPPH and ORAC of the orange samples is also presented in Figure 1. Values are expressed in mM TE/kg of fruit. The DPPH assay is used to predict antioxidant activities according to the mechanism by which antioxidants act to inhibit lipid oxidation. While the ORAC assay is used to determine the substance’s ability to react with reactive oxygen species.

### 2.2. Effects of Increasing Concentrations of HE or OE on the Viability of Hepa-RG and LX-2 Liver Cells

**Figure 2:**
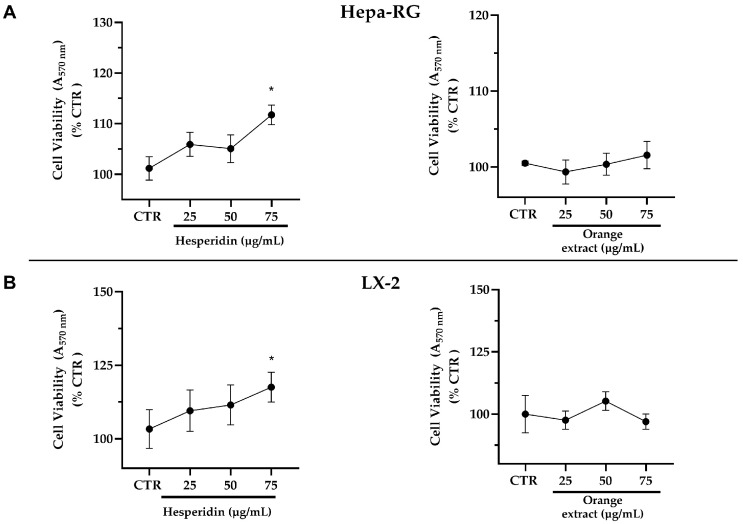
Effects of HE or OE on cell viability of Hepa-RG (**A**) and LX-2 (**B**) cells treated with increasing concentrations or its solvents (CTR) for 48 h. All data reported in each panel are expressed as the mean ± SEM from three independent experiments. Values are expressed as % cell viability compared to CTR. Statistical analyses: one-way ANOVA corrected for multiple comparison by Dunnett’s post-hoc analysis to compare differences between control and treated cells. * *p* ≤ 0.033.

The cytotoxic effects of HE or OE against human Hepa-RG and LX-2 liver cells were evaluated by MTT assay and are described in Figure 2. The data indicate that HE and OE, administered at increasing doses of 25, 50, and 75 µg/mL, exert no inhibitory effects on the growth of Hepa-RG and LX-2 cells at 48 h; rather, HE in both cell lines leads to a slight significant increase in viability at the higher dose. Also, no effect on cell vitality was observed at 24 h of treatment. This suggests that within the tested range, neither hesperidin nor the mixture of polyphenols in orange extract demonstrate dose-dependent cytotoxicity.

### 2.3. Anti-Inflammatory and Anti-Fibrotic Effects in Hepa-RG Cells Treated with HE or OE

**Figure 3:**
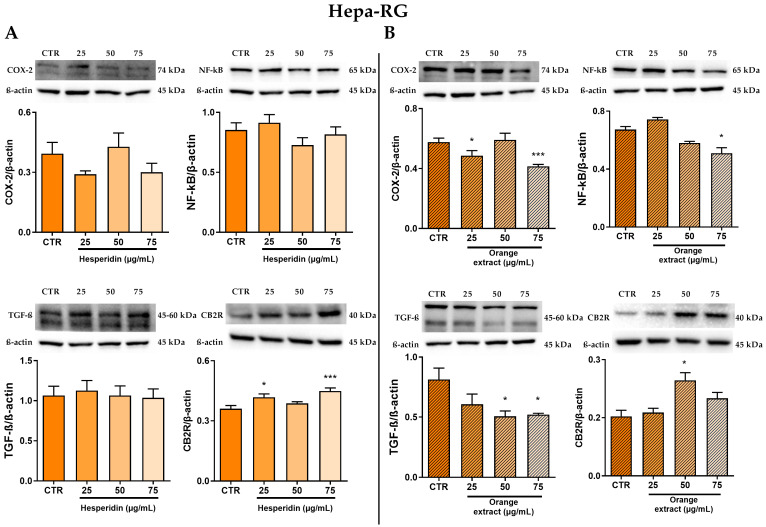
Effect of HE (**A**) and OE (**B**) on COX-2, NF-κB p65, TGF-β, and CB2R protein expressions in Hepa-RG cells was measured by Western blot, and β-actin was used as a loading control. Representative Western blot bands are shown below. All data reported in each panel are expressed as the mean ± SEM from three independent experiments, where * *p* ≤ 0.033 and *** *p* < 0.0002 by the Kruskal–Wallis test corrected for multiple comparison by Dunn’s test.

As shown in Figure 3A, following 48-h treatment with HE on Hepa-RG cells, the in vitro anti-inflammatory activity of HE was monitored by assessing the protein expression levels of the inflammation-related enzyme COX-2 and examining its modulation by the NF-κB transcription factor p65. There were no statistically significant changes in either target analyzed, although Western blot analysis showed a slight reduction in COX-2 protein levels after HE treatment.

Next, we evaluated the antifibrotic role of HE flavonoid by analyzing its effect on the protein expression of TGF-β, a key mediator of fibrosis. However, our results revealed that TGF-β expression did not change significantly with HE treatment.

HE resulted in a slight, but statistically significant, increase in CB2R protein expression from a concentration of 25 µg/mL of this flavonoid. Interestingly, we observed even higher levels of the cannabinoid type 2 receptor after treatment with 75 µg/mL HE than in the control.

Furthermore, we performed Western blot experiments to analyze the same inflammatory and fibrotic targets in Hepa-RG cells, in this case using OE rather than the single flavonoid HE with the same increasing concentrations and for the same incubation time (48 h).

In Figure 3B, the WB analysis shows that the extract led to a significant reduction in the amount of NF-κB p65 protein only at a concentration of 75 µg/mL. It is noteworthy that a marked reduction in the expression levels of the pro-inflammatory enzyme COX-2 was observed, both at 25 µg/mL and even more markedly at 75 µg/mL.

In addition to the anti-inflammatory effects, treatment with the extract effectively inhibited protein expression of the pro-fibrotic agent TGF-β, demonstrating a dose-dependent response to increasing extract concentration.

Finally, the OE treatment resulted in an approximately 55% increase in CB2R protein levels compared to the control, particularly at a concentration of 50 µg/mL OE.

**Figure 4:**
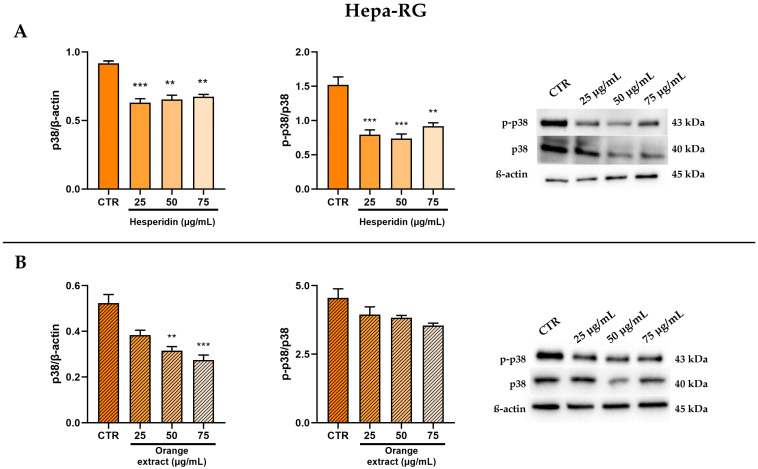
Effect of HE (**A**) and OE (**B**) on total and phosphorylated p38 MAPK protein expressions in Hepa-RG cells was measured by Western blot, and β-actin was used as a loading control. Representative Western blot bands are shown below. All data reported in each panel are expressed as the mean ± SEM from three independent experiments, where ** *p* ≤ 0.002, and *** *p* < 0.0002 by the Kruskal–Wallis test corrected for multiple comparisons by Dunn’s test.

Next, we examined the potential effect of HE on the coupling of the CB2R to p38 MAPK to understand its role in mediating anti-inflammatory effects. As expected, we observed that the protein expression of p38 MAPK was significantly reduced by about 30% compared to the control, in a statistically significant manner at all tested doses of HE. Furthermore, this significant reduction is also found for p-p38 expression levels and the p-p38/p38 ratio (Figure 4A).

The treatment with increasing concentrations of OE shows a significant reduction of the levels of p38 MAPK and its phosphorylated form (Figure 4B).

### 2.4. Anti-Inflammatory and Anti-Fibrotic Effects in LX-2 Cells Treated with HE or OE

**Figure 5:**
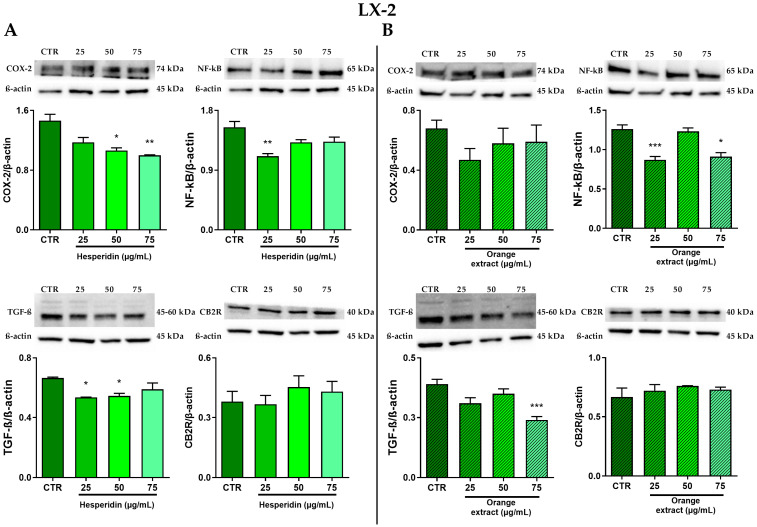
Effect of HE (**A**) and OE (**B**) on COX-2, NF-κB p65, TGF-β and CB2R protein expressions in LX-2 cells was measured by Western blot, and β-actin was used as a loading control. Representative Western blot bands are shown below. All data reported in each panel are expressed as the mean ± SEM from three independent experiments, where * *p* ≤ 0.033, ** *p* ≤ 0.002, and *** *p* < 0.0002 by the Kruskal–Wallis test corrected for multiple comparison by Dunn’s test.

To obtain a more complete understanding of the anti-inflammatory and antifibrotic effects of HE and OE, we also extended the Western blot analysis to hepatic LX-2 stellate cells. The results in Figure 5A showed that treatment with HE for 48 h resulted in lower levels of NF-κB p65 protein expression after stimulation with 25 µg/mL hesperidin compared to control. Notably, treatment with this flavonoid led to a significant decrease in the amount of COX-2, which was inversely proportional to the increase in HE doses.

Following HE treatment, LX-2 cells showed a significant reduction in TGF-β protein levels, particularly at 25 and 50 µg/mL, while this reduction is less pronounced at the highest dose of HE that was tested in these experiments.

In addition, treatment with HE slightly increased the amount of CB2R protein.

Western blot experiments were also conducted in LX-2 cells to verify the anti-inflammatory and antifibrotic effects of OE after 48 h of treatment (Figure 5B). While OE is able to effectively reduce the expression of NF-κB p65, with a significant decrease at 25 and 75 µg/mL, no significant modulation of COX-2 protein levels was evidenced. Orange extract is able to modulate protein levels of the pro-fibrotic cytokine TGF-β in a dose-dependent manner, whereas CB2R levels were not influenced by the 48-h treatment with the extract.

**Figure 6:**
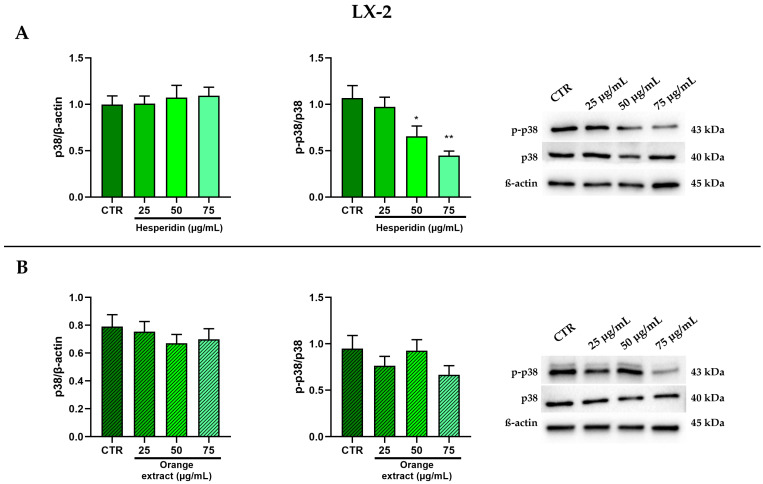
Effect of HE (**A**) and OE (**B**) on total and phosphorylated p38 MAPK protein expressions in LX-2 cells was measured by Western blot, and β-actin was used as a loading control. Representative Western blot bands are shown below. All data reported in each panel are expressed as the mean ± SEM from three independent experiments, where * *p* ≤ 0.033 and ** *p* ≤ 0.002 by the Kruskal–Wallis test corrected for multiple comparison by Dunn’s test.

As shown in Figure 6, in LX-2 cells, only HE treatment significantly reduced p38 MAPK phosphorylation after normalization to total p38.

### 2.5. Anti-Oxidant Effects of HE or OE Treatments

The antioxidant effect was evaluated by analyzing the protein levels of the antioxidant enzymes SOD1, SOD2, and CAT after the treatments with HE and OE.

**Figure 7:**
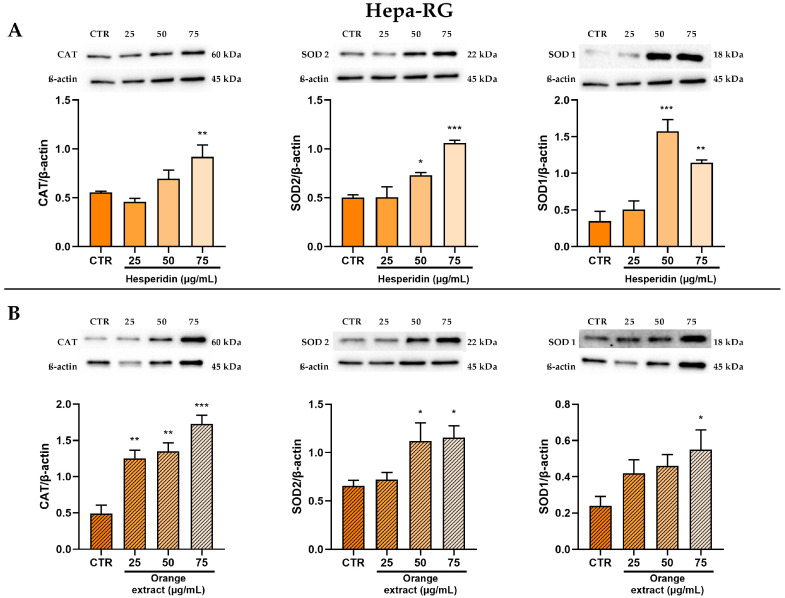
Effect of HE (**A**) and OE (**B**) on CAT, SOD2, and SOD1 protein expressions in Hepa-RG cells was measured by Western blot, and β-actin was used as a loading control. Representative Western blot bands are shown below. All data reported in each panel are expressed as the mean ± SEM from three independent experiments, where * *p* ≤ 0.033, ** *p* ≤ 0.002, and *** *p* < 0.0002 by the Kruskal–Wallis test corrected for multiple comparison by Dunn’s test.

As shown in Figure 7, both treatments in Hepa-RG cells led to a marked and significant increase in the protein levels of these key antioxidant enzymes, which was greater at the highest treatment concentrations.

**Figure 8:**
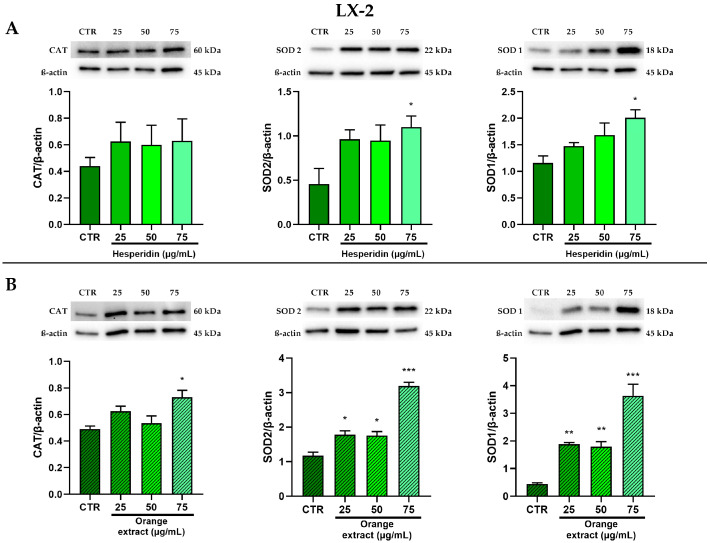
Effect of HE (**A**) and OE (**B**) on CAT, SOD2, and SOD1 protein expressions in LX-2 cells was measured by Western blot, and β-actin was used as a loading control. Representative Western blot bands are shown below. All data reported in each panel are expressed as the mean ± SEM from three independent experiments, where * *p* ≤ 0.033, ** *p* ≤ 0.002, and *** *p* < 0.0002 by the Kruskal–Wallis test corrected for multiple comparison by Dunn’s test.

LX-2 cells also showed a more pronounced significant increase in the protein levels of SOD1 and SOD2 following both treatments, than CAT protein levels, which still showed an increasing trend (Figure 8).

**Figure 9:**
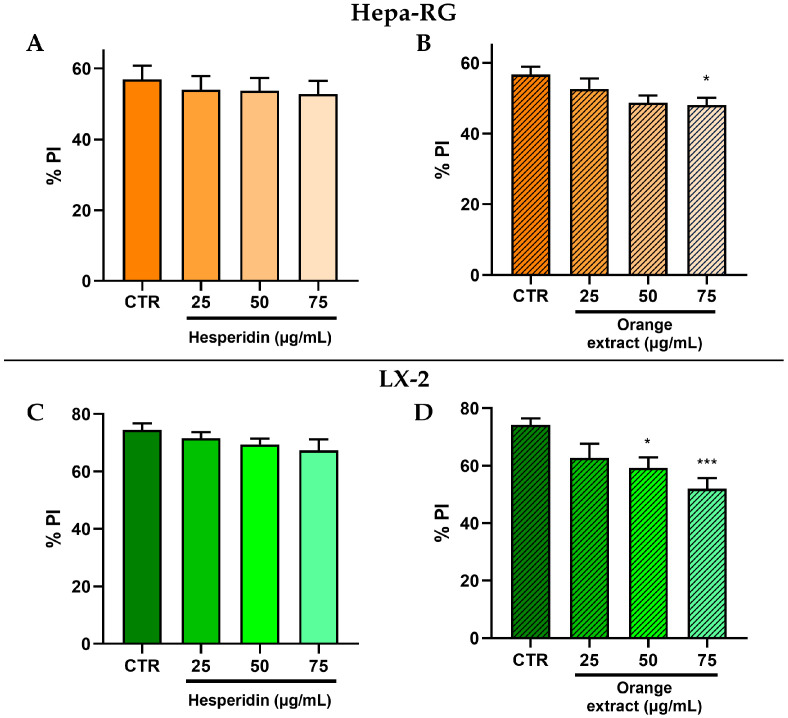
Peroxidation index (PI) levels in Hepa-RG cells treated with HE (**A**) and OE (**B**); PI levels in LX-2 cells treated with HE (**C**) and OE (**D**). All data reported in each panel are expressed as the mean ± SEM from three independent experiments, where * *p* ≤ 0.033 and *** *p* < 0.001 by one-way ANOVA corrected for multiple comparison by Dunnett’s *post hoc* analysis.

To further investigate the effects of HE and OE at different concentrations, we analyzed the lipid composition and peroxidation of cell membranes. Using lipidomic analysis, we determined the PI levels after 48 h of treatment in Hepa-RG and LX-2 cells (Figure 9). Compared to the control group, in Hepa-RG cells, the treatment with OE reduced PI only at 75 μg/mL in a significant manner (Figure 9B). While in LX-2 cells treated with OE, a significant reduction of PI was observed at 50 μg/mL and 75 μg/mL (Figure 9D).

This decrease is mainly due to the contribution of polyunsaturated fatty acids (PUFAs) and, in particular, the significant decrease of omega-6 PUFAs such as linoleic acid (LA) and arachidonic acid (AA). On the contrary, an increase of saturated fatty acids (SFAs) was detected, especially in LX-2 cells (Table 1).

### 2.6. Anti-Fibrotic Effects of HE and OE Treatments in LX-2-Activated Cells

**Figure 10:**
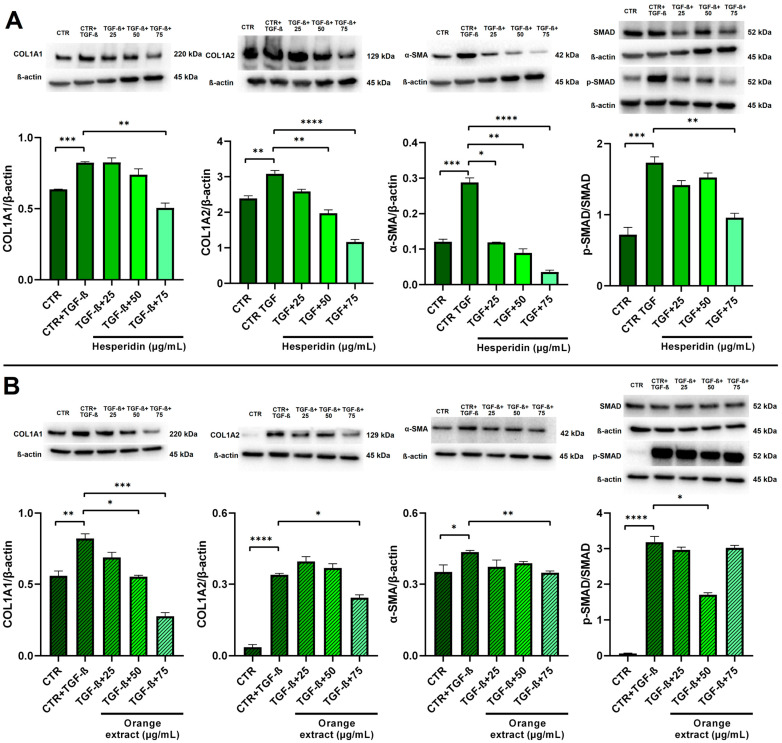
Effect of HE (**A**) and OE (**B**) on COL1A1, COL1A2, and α-SMA protein expressions and the p-SMAD/SMAD ratio in LX-2-activated cells was measured by Western blot, and β-actin was used as a loading control. Representative Western blot bands are shown below. All data reported in each panel are expressed as the mean ± SEM from three independent experiments, where * *p* ≤ 0.033, ** *p* ≤ 0.002, *** *p* < 0.0002, and **** *p* < 0.0001 by the Kruskal–Wallis test corrected for multiple comparisons by Dunn’s test.

Activated LX-2 cells were used as an in vitro model to investigate the potential anti-fibrotic effects of HE and OE. TGF-β (5 ng/mL) was employed as a prototypical stimulus to induce LX-2 cell activation. As shown in Figure 10, TGF-β significantly upregulated the expression of key liver fibrogenesis markers, including α-smooth muscle actin (αSMA), collagen type I alpha 1 chain (COL1A1), and collagen type I alpha 2 chain (COL1A2). In fibrotic livers, the TGF-β-mediated signaling pathway depends on the phosphorylation of mothers against decapapentaplegic (SMAD) 2/3 [33]. In our study, we confirmed that activation of LX-2 cells led to a significant increase in TGF-β expression and phosphorylated SMAD 2/3.

Western blot analysis revealed that HE treatment effectively reduced the expression levels of COL1A2 and α-SMA in a dose-dependent manner during TGF-β-induced LX-2 cell activation. However, it is noteworthy that only the higher concentration of HE (75 µg/mL) significantly counteracted the increase in COL1A1 expression. In contrast, OE exhibited a more potent anti-fibrotic effect, inhibiting the increase in COL1A1 even at lower concentrations compared to HE. However, higher concentrations of OE were required to significantly reduce α-SMA and COL1A2.

To further explore the mechanisms underlying the effects of HE and OE, we examined the involvement of the TGF-β/SMAD pathway. Protein expression of SMAD 2/3 was assessed, revealing a significant increase in phosphorylation following TGF-β stimulation. Notably, both HE at 75 µg/mL and OE at 50 µg/mL effectively reduced TGF-β-induced phosphorylation, suggesting that these compounds may modulate the TGF-β/SMAD signaling pathway.

## 3. Discussion

Several studies have shown that the chronic liver inflammation results in a fibrotic state due to the triggering of tissue repair mechanisms [34,35,36]. Pro-inflammatory mediators that are generated as a result of cell damage induce transdifferentiation of mesenchymal precursor cells into myofibroblast-like cells, which release pro-inflammatory and pro-fibrogenic factors including TGF-β [14]. Hepatic stellate cells then trigger fibrogenic activation by phagocytosing damaged hepatocytes, transdifferentiating, and expressing mesenchymal cell markers such a αSMA and type I collagen [37]. Therefore, it is suggested that hepatic stellate cells are primarily responsible for the establishment and maintenance of fibrosis. A cause-and-effect relationship correlating the inflammatory state and the development of fibrosis is thus shown [38].

Several studies [12,39,40,41] emphasize an anti-inflammatory effect of polyphenols on liver cells. An experimental study demonstrated the ability of resveratrol, a polyphenol contained in red wine, grape skin and other vegetables, to inhibit NF-κB in a mouse model and an LX-2 cell model of liver fibrosis [42]. Treatment of HSC-T6 cells with apple-derived polyphenols has been shown to reduce activation of stellate cells and the TLR4/NF-κB/TGF-β signaling pathway by inhibiting liver inflammation and subsequent fibrosis [43]. In addition, the treatment with a polyphenol-enriched fraction (PEF) from Acalypha wilkesiana was seen to reduce the expression of proinflammatory cytokines and the COX-2 enzyme in RAW 264.7 macrophages and a mouse model of liver damage, associated with the activation of MAPKs, p38, and consequently NF-κB [44]. Analogous results were also obtained after treatment of the same cell line with polyphenols extracted from Hibiscus sabdariffa Linnaeus, revealing the anti-inflammatory potency of polyphenols [45].

The modulation of inflammatory pathways by hesperidin has also been detected in experimental models, focusing on the NF-κB/COX-2/p38 MAPK-directed pathway [46].

In light of the ability of HE to modulate the hepatic inflammatory response and to prevent liver fibrosis, our results showed that cellular treatments with HE and OE tend to have an effect on TGF-β protein expression, delineating their ability to influence the fibrotic signaling pathways.

TGF-β is central in chronic liver disease, contributing to all stages of disease progression from initial liver injury through inflammation and fibrosis to cirrhosis and hepatocellular carcinoma [47,48,49].

The effects of HE and OE on liver fibrosis were evaluated using an in vitro model of TGF-β-activated LX-2 cells, which mimic the activation of hepatic stellate cells [50]. Our results show that both HE and OE significantly inhibit TGF-β-induced fibrosis markers such as α-SMA, COL1A1, and COL1A2. Furthermore, the reduction in SMAD2/3 phosphorylation following treatment with HE and OE suggests that these compounds may exert their antifibrotic effects by interfering with the TGF-β/SMAD signaling pathway, thereby preventing the transcriptional activation of genes responsible for fibrotic matrix accumulation.

It is known in the literature how the CB2R can reduce inflammation and fibrosis by promoting differentiation into the M2 anti-inflammatory phenotype at the expense of the M1 pro-inflammatory phenotype of Kupffer cells resulting in reduced release of pro-inflammatory cytokines [31,51,52]. In a mouse model of alcoholic liver disease, the absence of CB2R in the liver exacerbated hepatic steatosis [53]. Moreover, CB2R regulates liver development and metabolism as demonstrated in zebrafish, where CB2R absence resulted into the development of a smaller liver and a reduced hepatocytes differentiation [54]. Considering its dysregulation in various pathologies [55] and given the CB2R involvement in several conditions such as adipose tissue inflammation, insulin sensitivity, and glucose metabolism, to date, the endocannabinoids system has become a promising target for the study of numerous diseases such as metabolic syndrome, obesity, and other lifestyle-related diseases [56,57].

Therefore, molecules able to modulate this system are being explored, which could mimic the action of endogenous ligands to promote the expression of cannabinoid receptors [58,59].

In this study, given the increased expression of the CB2R after cell treatments, we investigated the downstream response, evaluating some inflammatory factors, such as p38 and c-Jun N-terminal kinases (JNK) MAPK, NF-κB p65 and COX-2. A down-regulation of p38 MAPK was found, due, probably, to the anti-inflammatory effect of CB2R. Indeed, p38 kinase has been shown in previous studies to play an important role in contributing to the progression of hepatic inflammation [60]. To better understand the anti-inflammatory effects of the treatments, we explored their influence on the activation of p38 MAPK, an enzyme that regulates pro-inflammatory factors. We evaluated the phosphorylation levels of this kinase, compared to the total enzyme levels, and a reduction in the p-p38/p38 ratio was observed. This effect appears to be significant only following the treatment with HE; however, the treatment with OE maintains a similar trend. These findings may suggest that treatments with polyphenols can reduce pro-inflammatory pathways in the cells.

Similarly, we found a down-regulation of NF-κB p65 and COX-2, factors known to have a pro-inflammatory effect. NF-κB plays a central role in the modulation of the inflammatory response. Likely, the activation of NF-κB leads to the transdifferentiation of hepatic stellate cells into myofibroblasts, resulting in the fibrogenic response [61].

NF-κB factor acts by controlling the activity of several inflammatory factors, including COX-2. Cyclooxygenases play a key role in the synthesis of prostaglandin E2 from arachidonic acid. COX-2 is a potent enzyme that can initiate the inflammation and promote prostaglandin synthesis. In addition to NF-κB, p38 MAPK can also regulate the expression of COX-2 [62].

The anti-inflammatory effect is probably based on two pathways of action: NF-κB → COX-2 and p38 MAPK → COX-2, which may be initiated by stimulation of the CB2R by polyphenols.

Given the well-known antioxidant properties of natural compounds and their influence on inflammatory mechanisms [23,63], we aimed to investigate this aspect in our cellular models following treatments with HE and OE.

The antioxidant capacity of OE, verified by DPPH and ORAC tests, was also confirmed at the cellular level in liver cells. Protein expression analyses revealed that such treatment increases the protein levels of SOD1, SOD2, and CAT, which are key enzymes in the cellular antioxidant response. These effects are also observed following treatment with HE, identified by phytochemical analysis as the main component of the extract.

Lipids are a major target of oxidative stress due to their abundance and susceptibility to oxidation. Among lipids, polyunsaturated fatty acids (PUFAs) are involved in secretion, fat storage, and lipid peroxidation [64]. In previous studies, we demonstrated the ability of polyphenols to modify the fatty acid profile of the cell membrane [65].

Lipid peroxidation, triggered by oxidative stress, activates inflammatory pathways that enhance the production of reactive oxygen species (ROS). The literature indicates that, under pathological conditions, lipid peroxidation is a cellular mechanism for responding to oxidative stress. The fatty acids most susceptible to peroxidation are those with a high number of double bonds, such as the polyunsaturated fatty acids (PUFAs) found in cell membranes [66].

This study demonstrates that polyphenols exert their antioxidant effects by acting at multiple levels, such as regulating key enzymes involved in the antioxidant response and modulating the membrane lipid profile, specifically by reducing membrane peroxidation levels.

Finally, the antioxidant properties of OE are also reflected in its anti-inflammatory effects, as demonstrated by the regulation of protein expression of key targets involved in the inflammatory process.

## 4. Materials and Methods

### 4.1. Preparation of the Extract of OE and Determination of the Total Phenolic Content

This study used Navelina oranges, a seedless blond citrus cultivar grown on an organic farm in Calabria, Italy. The orange samples were harvested when ripe in January 2023. Ten kg of oranges were sent to the Council for Agricultural Research and Economics (CREA), Turi, BA, Italy, for physicochemical analysis, determination of total polyphenols, and antioxidant activity (Figure 1) [67]. For the preparation of the OE, the fruit material was weighed and oven-dried at 37 °C, and 8 g of dry pulp were resuspended in 20 mL of a solution containing absolute ethanol: water:water:37% hydrochloric acid (70:30:1 v/v/v). The mixture was kept in the dark for 24 h, then centrifuged, and the supernatant was recovered and concentrated in a SpeedVac concentrator (Savant^®^SPD131DDA, Thermo Fisher Scientific, Waltham, MA, USA). The total phenolic profiles were determined using the Folin-Ciocalteu protocol with some modifications [68] and analyzed by ultra-high performance liquid chromatography/quadrupole time-of-flight mass spectrometry (UHPLC/QTOF) [69].

### 4.2. Cell Lines, Culture Media and Treatments

Human hepatoma cells (Hepa-RG) (Cat. n. HPRGC10) were purchased from Thermo Fisher Scientific (Waltham, MA, USA) and cultured in hepatocyte bullet kit medium (HBM, Thermo Fisher Scientific, Waltham, MA, USA). Human hepatic stellate cell line LX-2 (Cat. n. SCC064) was purchased from Millipore, (Merck Life Science, Milan, Italy) and cultured in Dulbecco’s modified Eagle’s medium (DMEM; Thermo Fisher Scientific, Milan, Italy). Each medium was supplemented with 10% heat-inactivated fetal bovine serum (FBS) (Thermo Fisher Scientific, Waltham, MA, USA) and 1% of antibiotic-antimycotic (10,000 units/mL of penicillin, 10,000 μg/mL of streptomycin, and 25 μg/mL of Gibco Amphotericin B penicillin) (Thermo Fisher Scientific, Waltham, MA, USA). Cells were routinely propagated and cultured in a cell culture incubator at 37 °C and 5% CO_2_.

Cells were expanded in a 6-well culture plate until the desired confluence was obtained (about 80%), then the culture medium was removed and replaced with medium added with three different concentrations of HE (purchased from Sigma-Aldrich, H5254, St. Louis, MO, USA) or OE: 25 µg/mL, 50 µg/mL and 75 µg/mL.

For OE (supplied by CREA) treatments from a 400,000 µg/mL stock solution provided by the OE manufacturers, an intermediate dilution of 10,000 µg/mL was prepared by diluting with 10% ethanol as suggested by the OE manufacturer. The control sample was added with 10% ethanol in an amount equal to the maximum amount of solvent present in the highest treatment concentration (0.75%). For HE treatments, a 10,000 µg/mL stock solution was prepared (100 mg powder in 10 mL DMSO). The control sample was added with DMSO in an amount equal to the maximum amount of solvent present at the highest treatment concentration (0.75%). For both OE and HE, treatment concentrations (25, 50, 75 µg/mL) were prepared from the 10,000 µg/mL concentration by diluting with the appropriate culture media.

The cells were then incubated for 48 h in the presence of the two treatments, at 37 °C and 5% CO_2_. After 48 h cells were scraped with PBS 1x and pelletted in microcentrifuge tubes to perform Western blot analysis.

For the activation of LX-2 cells with TGF-β, they were expanded in a 6-well culture plate until the desired confluence (about 80%) was reached. Then, the culture medium was removed and replaced with medium containing three different concentrations of HE or OE (25, 50, and 75 µg/mL) for a 2-h pre-treatment. Subsequently, a concentration of 5 ng/mL of TGF-β was added directly to the wells. After 24 h, the medium with TGF-β was removed and replaced with medium containing the three different concentrations of HE or OE treatments. The cells were then incubated for an additional 24 h in the presence of the two treatments. Next, the cells were scraped with PBS 1x and pelleted in microcentrifuge tubes for Western blot analysis.

For TGF-β (TGF beta 1/TGFB1 Protein, Human (HEK293), Cat. No.: HY-P70543, MedChemExpress, Monmouth Junction, NJ, USA), a 100 µg/mL stock solution was prepared by dissolving the powder in the solvent provided by the manufacturer. Then, an intermediate dilution of 5 µg/mL was prepared using DMEM.

### 4.3. Viability Assay and Western Blot Analysis

Cell viability was evaluated by the 3-[4,5-dimethylthiazol-2-yl]-2,5 diphenyl tetrazolium bromide (MTT) test (purchased from Sigma-Aldrich, St. Louis, MO, USA). Determination of cell growth was performed using the MTT assay at 48 h. The test is based on the assessment of the activity of mitochondrial dehydrogenases, active only in living cells, which reduce MTT to formazan. After the treatments, MTT (5 mg/mL) was added to each well at a volume of one-tenth of the original culture volume. After 3 h of incubation at 37 °C, protected from the light, the supernatant was removed. The formazan crystals were solubilized using acidic isopropanol (absolute isopropanol + 5% HCl). Then the absorbance values at 570 nm were determined.

Cells were lysed in RIPA buffer (Sigma-Aldrich, St. Louis, MO, USA) supplemented with the Halt Protease and Phosphatase Inhibitor (ThermoFisher Scientific, Waltham, MA, USA). The lysate was recovered, centrifuged at 14,000 rpm for 30 min at 4 °C, and the supernatant was collected and used for total protein quatification by a standard Bradford assay (Bio-Rad Laboratories, San Francisco, CA, USA). An equal amount of protein (30 μg) was separated in 4–15% Tris-glycine sodium dodecyl sulfate-polyacrylamide gel (Bio-Rad Laboratories, San Francisco, CA, USA). Membranes were incubated, with gentle shaking, with the following primary antibodies: CB2R (Anti-Cannabinoid Receptor II antibody, ab3561, 1:500, abcam, Cambridge, UK), p38 (p38α MAPK antibody, #9218, 1:500, Cell Signaling Technology, Danvers, MA, USA), p-p38 (Phospho-p38 MAPK (Thr180/Tyr182) (3D7) antibody, #9215, 1:500, Cell Signaling Technology), COX-2 (COX2 (D5H5) antibody, #12282, 1:500, Cell Signaling), NF-κB p65 (NF-κB p65 (D14E12) antibody, #8242, 1:500, Cell Signaling), TGF-β1 (TGF-β (56E4) antibody, #3709, 1:500, Cell Signaling), SOD1 (SOD1 (71G8) antibody, #4266, 1:6000, Cell Signaling), SOD2 (SOD2 (D3X8F) antibody, #13141, 1:6000, Cell Signaling), CAT (Catalase (D4P7B) antibody, #12980, 1:6000, Cell Signaling), COL1A1 (COL1A1 (E8I9Z) Rabbit mAb #91144, 1:500, Cell Signaling), COL1A2 (Anti-COL1A2 antibody, ab96723, 1:500, abcam), SMAD (SMAD2/3 Antibody #3102, 1:500, Cell Signaling) p-SMAD (Phospho-SMAD2 (Ser465/467)/SMAD3 (Ser423/425) (D27F4) Rabbit mAb #8828, 1:500, Cell Signaling), α-SMA (α-Smooth Muscle Actin (D4K9N) Rabbit mAb #19245, 1.500, Cell Signaling).

CB2R, COX-2, NF-κB p65, SOD1, SOD2, CAT, COL1A1, COL1A2 and p-SMAD antibodies were diluted in 5% *w*/*v* nonfat dry milk, 1X TBS, and 0.1% Tween^®^ 20. p38, p-p38, TGF-β, SMAD and α-SMA antibodies were diluted in 5% *w*/*v* BSA, 1X TBS, and 0.1% Tween^®^ 20. Experiments were carried out in triplicate. After overnight incubation of primary antibodies, anti-rabbit or anti-mouse secondary antibody (1:5000 Bio-Rad Laboratories, San Francisco, CA, USA), diluted in 5% *w*/*v* nonfat dry milk, 1X TBS, and 0.1% Tween^®^ 20, was incubated for one hour with gentle shaking. The chemiluminescence signal from proteins was revealed using Clarity Western ECL Substrate or Clarity Max Western ECL Substrate (Bio-Rad Laboratories, San Francisco, CA, USA) and analyzed using the chemiluminescence detection system ChemiDoc XRS (Bio-Rad Laboratories, San Francisco, CA, USA). The relative density of the bands was calculated using the ImageLab software 5.2.1 and the proteins detected were normalized against β-actin signal (β-Actin (13E5) Rabbit mAb #4970, 1:1000, Cell Signaling). The bar graphs were generated using the GraphPad 8.0.1 software.

### 4.4. Extraction of Lipids for Fatty Acid Analysis by Gas Chromatography

Lipid extraction was performed on Hepa-RG and LX-2 cell pellets treated with HE and OE at concentrations of 25, 50, and 75 μg/mL for 48 h, with untreated cells as the control. A modified Folch extraction method was used [70].

Briefly, a 100 µL aliquot of cell lysate was diluted with 450 µL of an acidified saline solution (2 × 10^−4^ M H_2_SO_4_, 0.1% NaCl). Subsequently, 2250 μL of a 2:1 chloroform:methanol mixture (Sigma-Aldrich, St. Louis, MO, USA) was added, the samples were vortexed and centrifuged. The lower phase, containing fatty acids, was collected and dried using a centrifugal evaporator (Thermo Fisher Scientific, Waltham, MA, USA). To obtain fatty acid methyl esters (FAME), 250 μL of toluene and 750 μL of boron trifluoride in 14% methanol (Sigma-Aldrich, St. Louis, MO, USA) were added and the mixture was incubated at 80 °C in a thermoblock (Thermo Fisher Scientific, Waltham, MA, USA) for 2 h. After cooling to room temperature for 10 min, 1250 μL of 5% NaCl and 250 μL of toluene (Sigma-Aldrich, St. Louis, MO, USA) were added and centrifuged at 700 g for 10 min at 4 °C. The upper phase containing FAME was collected and analyzed using a gas chromatograph with an autosampler, a split/splitless injector, an FID detector and a gaseous hydrogen generator (Thermo Fisher Scientific, Waltham, MA, USA). 1 μL of solution was injected in splitless mode (split flow rate 50 mL min^−1^, splitless time 1 min) into an SGE Analytical Science BPX 70 capillary column, P/N SGE054623, 60 m × 0.25 mm ID, BPX70 0.25 UM (SGE Europe Ltd., Milton Keynes, UK).

Quantification of fatty acid methyl esters was performed with ChromQuest 4.1 software (Thermo Fisher Scientific, Focus GC, Waltham, MA, USA) using a standard mix (Supelco 37-Component FAME Mix, Sigma-Aldrich, St. Louis, MO, USA). Of the 37 fatty acids (FAs) analyzed, we paid attention to the three main FA families: saturated fatty acids (SFAs), monounsaturated fatty acids (MUFAs) and polyunsaturated fatty acids (PUFAs). Specifically, we concentrated on omega-6 PUFAs such as linoleic acid (LA), arachidonic acid (AA), and dihomo-gamma-linolenic acid (DGLA) and omega-3 PUFAs such as eicosapentaenoic acid (EPA), and docosahexaenoic acid (DHA). Taking into account the mentioned FAs, the Peroxidation Index (PI) was calculated [(% MUFAs × 0.025) + (% LA × 1) + (% DGLA × 2) + (% AA × 4) + (% EPA × 6) + (% DHA × 8)] [66].

### 4.5. Statistical Analysis

One-way ANOVA corrected for multiple comparison by Dunnett’s post-hoc analysis was performed to compare differences in cell viability and lipidomic profiles, between control and treated cells. Kruskal-Wallis test corrected for multiple comparison by Dunn’s test was performed to test differences in protein expression between control and treated cells. Statistical significance was set at * *p* ≤ 0.033, ** *p* ≤ 0.002, and *** *p* < 0.0015.

## 5. Conclusions

In summary, our study highlights the potential of hesperidin and Navelina orange extract as crucial modulators of inflammation and liver fibrosis through their significant effects on key inflammatory pathways, such as NF-κB/COX-2 and p38 MAPK/COX-2.

OE and HE effectively reduce the expression of essential fibrosis markers and may influence the fibrotic response by inhibiting downstream signaling events involved in fibrogenesis.

Simultaneously, these effects seem be modulated by CB2R expression, leading to a significant reduction of hepatic fibrosis by impacting TGF-β cytokine levels in both hepatocytes and stellate cells. Furthermore, these treatments promoted the antioxidant response in cells by acting on several processes. Future studies will investigate the action of such treatments on activated hepatic stellate cells, on which we expect a greater anti-inflammatory effect, already significant on nonactivated cells.

## Figures and Tables

**Table 1:** (A) Mean percentage of n-6 and n-3 PUFAs and total fatty acid in the Hepa-RG membrane cells treated with increasing concentrations of HE and OE (25, 50, and 75 μg/mL) after 48 h of treatment; (B) Mean percentage of n-6 and n-3 PUFAs and total fatty acid in the LX-2 membrane cells treated with increasing concentrations of HE and OE (25, 50, and 75 μg/mL) after 48 h of treatment.

**A** **Hepa-RG Fatty Acids (%)**	**HE**				**OE**			
	**CTR**	**25 µg/mL**	**50 µg/mL**	**75 µg/mL**	**CTR**	**25 µg/mL**	**50 µg/mL**	**75 µg/mL**
n-6 PUFAs								
LA	3.83 ± 0.06	3.66 ± 0.18	3.53 ± 0.20	3.66 ± 0.19	2.97 ± 0.06	3.07 ± 0.05	2.92 ± 0.14	2.59 ± 0.06 *
DGLA	1.02 ± 0.06	0.97 ± 0.05	1.03 ± 0.03	0.93 ± 0.06	0.79 ± 0.10	0.62 ± 0.09	0.61 ± 0.03	0.52 ± 0.07
AA	5.38 ± 0.28	4.65 ± 0.17	5.06 ± 0.12	4.68 ± 0.25	5.45 ± 0.12	5.65 ± 0.32	4.59 ± 0.08 *	4.80 ± 0.17
n-3 PUFAs								
EPA	1.01 ± 0.04	1.13 ± 0.07	1.18 ± 0.09	1.02 ± 0.04	0.86 ± 0.05	0.69 ± 0.1	0.67 ± 0.01	0.67 ± 0.02
DHA	2.86 ± 0.19	2.8 ± 0.16	2.52 ± 0.11	2.72 ± 0.14	3.06 ± 0.12	2.61 ± 0.3	2.69 ± 0.09	2.57 ± 0.11
Total fatty acids								
SFAs	56.98 ± 0.61	55.92 ± 1.83	57.67 ± 0.44	58.24 ± 1.93	47.41 ± 5.28	48.27 ± 4.1	48.95 ± 1.26	49.12 ± 2.2
MUFAs	26.03 ± 0.12	27.59 ± 1.4	25.39 ± 0.26	25.61 ± 1.59	26.9 ± 0.36	28.07 ± 1.18	26.4 ± 0.58	25.64 ± 1.29
PUFAs	16.99 ± 0.70	16.49 ± 0.43	16.95 ± 0.35	16.15 ± 0.4	16.42 ± 0.44	15.31 ± 1.18	14.27 ± 0.18	13.56 ± 0.52 *
**B** **LX-2 Fatty Acids (%)**	**HE**				**OE**			
	**CTR**	**25 µg/mL**	**50 µg/mL**	**75 µg/mL**	**CTR**	**25 µg/mL**	**50 µg/mL**	**75 µg/mL**
n-6 PUFAs								
LA	2.92 ± 0.04	2.49 ± 0.18	2.51 ± 0.20	2.74 ± 0.04	2.28 ± 0.09	2.29 ± 0.23	2.36 ± 0.2	2.53 ± 0.21
DGLA	1.14 ± 0.04	1.11 ± 0.01	1.11 ± 0.03	0.99 ± 0.10	1.07 ± 0.08	0.96 ± 0.17	0.90 ± 0.13	0.90 ± 0.19
AA	7.29 ± 0.13	7.25 ± 0.10	6.99 ± 0.02	6.45 ± 0.23 **	7.40 ± 0.16	6.16 ± 0.88	5.69 ± 0.75	5.44 ± 1.19
n-3 PUFAs								
EPA	0.79 ± 0.07	0.68 ± 0.03	0.58 ± 0.04	0.66 ± 0.04	0.55 ± 0.08	0.61 ± 0.13	0.52 ± 0.08	0.59 ± 0.19
DHA	4.31 ± 0.14	4.12 ± 0.12	4.05 ± 0.05	4.01 ± 0.19	4.50 ± 0.18	3.69 ± 0.59	3.57 ± 0.41	2.70 ± 0.12
Total fatty acids								
SFAs	47.41 ± 1.44	47.26 ± 1.19	48.39 ± 0.60	49.55 ± 0.10	47.51 ± 1.11	53.44 ± 6.11	57.14 ± 5.23	59.45 ± 5.96 *
MUFAs	33.91 ± 1.05	34.43 ± 0.99	33.82 ± 0.76	31.99 ± 0.19	33.79 ± 0.76	31.14 ± 3.99	28.01 ± 3.64	26.84 ± 3.87
PUFAs	18.68 ± 0.40	18.31 ± 0.24	17.79 ± 0.35	18.46 ± 0.16	18.69 ± 0.47	15.42 ± 2.15	14.86 ± 1.6	13.72 ± 2.08

All data are expressed as the mean ± SEM from three independent experiments. The *p*-value was determined by one-way ANOVA corrected for multiple comparisons by Dunnett’s post hoc analysis. * *p* ≤ 0.033 and ** *p* ≤ 0.002. Abbreviations: LA, linoleic acid; DGLA, dihomo-gamma-linolenic acid; AA, arachidonic acid; EPA, eicosapentaenoic acid; DHA, docosahexaenoic acid; SFAs, saturated fatty acids; MUFAs, monounsaturated fatty acids; PUFAs, polyunsaturated fatty acids.

## Data Availability

Data are available from the corresponding author upon reasonable request.
